# Inhibitors of ubiquitin E3 ligase as potential new antimalarial drug leads

**DOI:** 10.1186/s40360-017-0147-4

**Published:** 2017-06-02

**Authors:** Jagrati Jain, Surendra K. Jain, Larry A. Walker, Babu L. Tekwani

**Affiliations:** 1 National Center for Natural Products Research, Research Institute of Pharmaceutical Sciences, School of Pharmacy, University of Mississippi, Oxford, MS USA; 2 Department of BioMolecular Sciences, Division of Pharmacology, School of Pharmacy, University of Mississippi, Oxford, MS USA

**Keywords:** Malaria, *Plasmodium falcip*arum, Ubiquitine, Proteasome, Ubiquitine E3 ligase, Antimalarial

## Abstract

**Background:**

Protein ubiquitylation is an important post-translational regulation, which has been shown to be necessary for life cycle progression and survival of *Plasmodium falciparum*. Ubiquitin is a highly conserved 76 amino acid polypeptide, which attaches covalently to target proteins through combined action of three classes of enzymes namely, the ubiquitin-activating enzyme (E1), ubiquitin-conjugating enzyme (E2) and ubiquitin-protein ligase (E3). Ubiquitin E1 and E2 are highly conserved within eukaryotes. However, the *P. falciparum* E3 ligase is substantially variable and divergent compared to the homologs from other eukaryotes, which make the E3 ligase a parasite-specific target.

**Methods:**

A set of selected E3 ubiquitin ligase inhibitors was tested in vitro against a chloroquine-sensitive *P. falciparum* D6 strain (*Pf*D6) and a chloroquine-resistant *P. falciparum* W2 strain (*Pf*W2). The inhibitors were also tested against Vero and transformed THP1 cells for cytotoxicity. The lead antimalarial E3 ubiquitin ligase inhibitors were further evaluated for the stage-specific antimalarial action and effects on cellular development of *P. falciparum* in vitro. Statistics analysis was done by two-way ANOVA followed by Tukey and Sidak multiple comparison test using GraphPad Prism 6.

**Results:**

E3 ligase inhibitors namely, JNJ 26854165, HLI 373 and Nutlin 3 showed prominent antimalarial activity against *Pf*D6 and *Pf*W2. These inhibitors were considerably less cytotoxic to mammalian Vero cells. JNJ 26854165, HLI 373 and Nutlin 3 blocked the development of *P. falciparum* parasite at the trophozoite and schizont stages, resulting in accumulation of distorted trophozoites and immature schizonts.

**Conclusions:**

Interruption of trophozoites and schizont maturation by the antimalarial E3 ligase inhibitors suggest the role of ubiquitin/proteasome functions in the intraerythrocytic development of malaria parasite. The ubiquitin/proteasome functions may be critical for schizont maturation. Further investigations on the lead E3 ligase inhibitors shall provide better understanding regarding the importance of E3 ligase functions in the malaria parasite as a potential new antimalarial drug target and a new class of antimalarial drug leads.

## Background

According to the most recent estimates of the World Health Organization (WHO), in 2015 alone malaria caused 438,000 deaths with estimated 214 million new cases [1]. Infections with *Plasmodium falciparum* are responsible for more than 90% of malaria-related deaths, particularly in African countries [1]. Drug resistance of *P. falciparum* is the greatest challenge in the fight against malaria, as the parasite has developed resistance to most of the drugs presently used for the treatment of malaria. These factors have led to the earnest need to discover new molecular targets and identify new drug leads against those targets [2].

Post-translational modifications are necessary for the progression of *P. falciparum* life cycle [3, 4]. Among these modifications, protein ubiquitylation plays an important role. Ubiquitylation of proteins can be proteasome-dependent or independent. Proteasome-dependent ubiquitylation pathway involves various biological processes comprising specific components, which make this pathway a potential therapeutic target to develop new anti-malarial drugs. Ubiquitin system participates in various cellular functions as well as host-parasite interactions [3]. Ubiquitin is a highly conserved 76 amino acids’ polypeptide that covalently attaches to target proteins through the combined action of three classes of enzymes namely, the ubiquitin-activating enzyme (E1), the ubiquitin-conjugating enzyme (E2) and the ubiquitin-protein ligase (E3) [5]. At the onset, the ubiquitin-activating enzyme (E1) activates ubiquitin through an ATP-dependent reaction. In this reaction, the terminal glycine residue of ubiquitin is adenylated and transferred to an internal cysteine residue to generate a high-energy thiol ester intermediate. The activated ubiquitin is then transferred to the ubiquitin-conjugating enzyme (E2), forming another thiol-ether intermediate, which then specifically binds to the ubiquitin-protein ligase (E3). Lastly, E3 ligase transfers the activated ubiquitin from E2 to the side chain of a lysine residue of the target protein [3, 5]. Protein ubiquitylation can be monoubiquitylation (attachment of one ubiquitin), multiubiquitylation (several ubiquitin molecules attached to different lysine residues) or polyubiquitylation (polyubiquitin chain generation) [4].

Ubiquitin E1 and E2, the two enzymatic components of ubiquitylation system, are highly conserved within eukaryotes [4]. However, *P. falciparum* E3 ubiquitin ligases are considerably diverse and thus may serve as promising targets for new antimalarial discovery [5]. The hypothesis behind this study is that targeting the E3 ligases functions of ubiquitin system would selectively kill the malaria parasite and would be less toxic to the mammalian cells. A set of standard E3 ligase inhibitors was tested against the chloroquine (CQ)-sensitive *P. falciparum* D6 strain (*Pf*D6) and CQ-resistant *P. falciparum* W2 strain (*Pf*W2) *in vitro*. The selective antimalarial inhibitors were further investigated to analyze the effect of E3 ligase inhibitors on intraerythrocytic *P. falciparum* asexual life cycle development. These studies suggest ubiquitin E3 ligases as potential antimalarial drug targets and support previous reports that the ubiquitin proteosomal degradation pathway may be essential for the survival of the malarial parasite [4, 5].

## Methods

### Cell cultures

CQ-sensitive (D6, Sierra Leone) and -resistant (W2, Indochina) strains of *P. falciparum* cultures were obtained from Malaria Research and Reference Reagent Resource Center (MR4), USA and maintained at 5% hematocrit in complete culture medium (RPMI 1640 supplemented with 60 μg/mL amikacin, 27 mM NaHCO_3_ and 10% heat-inactivated normal human type A+ serum). The medium of the culture was replaced every 48 h, flushed with a gas mixture of 90% N2, 5% CO2 and 5% O2 and incubated at 37 °C.

Vero cells and THP1 cells were maintained in RPMI 1640 supplemented with 10% fetal bovine serum (FBS) at 37 °C in an incubator with 5% CO2. Both, Vero cells and THP1 cells were obtained from American Type Culture Collection (ATCC), USA having catalogue number ATCC® CCL-81™ and ATCC® TIB-202™ respectively and were subcultured twice a week.

### Chemicals

Eight E3 ligase inhibitors namely, thalidomide, proTAME, NSC 66811, Nutlin 3, HLI 373, JNJ 26854165, SMER 3 and NSC 146109 were used in the study. Thalidomide targets cereblon (CRBN), which makes an E3 ubiquitin ligase complex with damaged DNA-binding protein 1 (DDB1) and cullin-4A (Cul4A). Thalidomide binds with CRBN and inhibits the associated ubiquitin ligase activity [6, 7]. ProTAME, a cell-permeable prodrug, converts to an active molecule, tosyl-L-arginine methyl ester (TAME) and inhibits the ubiquitin ligase activity of the anaphase-promoting complex/cyclosome (APC/C) [8, 9]. NSC 66811 and Nutlin 3 have been reported as MDM2 (Murine Double Minute 2) E3 ligase inhibitors [10, 11]. JNJ 26854165 and HLI 373 are HDM2 (Human Double Minute 2) E3 ligase antagonists. SMER 3 is an inhibitor of a yeast SCF family E3 ubiquitin ligase (SCFMet30). Thalidomide, NSC6811, NSC 146109 and SMER 3 were purchased from Tocris Bioscience. Nutlin 3, JNJ 26854165 and ProTAME were purchased from Selleckchem, USA, Axon Medchem, Netherland and Boston Biochem, Inc. USA respectively. Chloroquine (CQ) was used as the positive control, and dimethyl sulfoxide (DMSO) was used as vehicle control in this study. CQ and DMSO were obtained from Sigma-Aldrich, USA.

### Antimalarial SYBR Green I-based fluorescence assay

CQ-sensitive *P. falciparum* D6 strain (*Pf* D6) and CQ-resistant *P. falciparum* W2 strain (*Pf* W2) were grown in RPMI 1640 medium supplemented with amikacin (60 μg/mL), NaHCO_3_ (27 mM) and 10% heat-inactivated normal human type A+ serum. Only ring stages of the parasite were counted and adjusted to 2% parasitemia with 2% hematocrit. Stock solutions (10 mM) of the test inhibitors were prepared in DMSO. The compounds were serially diluted in serum-free RPMI-1640 medium for testing at eight concentrations. The maximum concentrations of E3 ligase inhibitors and CQ used in the assay were 50 μM and 500 nM respectively. The plates were incubated at 37 °C in 5% CO_2_, 5% O_2_, and 90% N_2_ environment for 48, 72, 96 and 120 h. After incubation, SYBR green with RBC lysis buffer was added to each well, and the plates were incubated for 1 h in the dark at room temperature. The standard antimalarial agent, CQ was tested as the positive control, and DMSO was tested as vehicle control. Standard fluorescence was measured Fluostar Galaxy microplate reader (BMG Lab Technologies) at excitation wavelength of 485 nm and emission wavelength of 535 nm [12]. The anti-malarial activity (expressed as IC_50_ values) was determined by the dose-response analysis with XLfit©.

### In vitro cytotoxicity assays against Vero and transformed THP1 cells

The E3 ligase inhibitors were also tested against Vero cells (African green monkey kidney epithelial cells) and transformed non-dividing human monocytic (THP1) cells. The methods described earlier were used to measure cytotoxicity for Vero and THP1 cells [13, 14]. In brief, four days’ old Vero and THP1 cell cultures were diluted with RPMI medium to 25,000 cells/mL. Vero cells (100 μL per well) were seeded in a clear 96 well culture plate and incubated for 24 h at 37 °C in a 5% CO2 incubator to obtain confluent cells. Phorbol 12-myristate 13-acetate (PMA) (25 ng/mL) was added to THP1 cell culture to transform the cells to adherent macrophages [15]. PMA treated THP1cells were dispensed into clear 96 well culture plates (200 μL/well; 25,000 cells/mL) and incubated at 37 °C in a 5% CO_2_ incubator for overnight. The test compounds were prepared in DMSO at 10 mM concentration. The compounds were serially diluted in RPMI medium with 2% FBS in the separate daughter plates, for testing at eight concentrations. The cells were washed with culture medium, replaced with the fresh medium and test compounds were added. The maximum concentration of E3 ligase inhibitors and CQ used in these assays were 50 μM and 500 nM respectively. DMSO was tested as vehicle control in these assays. The plates were incubated further in a CO_2_ incubator at 37 °C for 48 h. After 48 h, 10 μL of AlamarBlue® was added to each well, and the plates were incubated in a CO_2_ incubator at 37 °C for overnight. Standard fluorescence was measured on a Fluostar Galaxy microplate reader (BMG Lab Technologies) at 544 nm ex, 590 nm em [13]. IC_50_ values were computed by analyzing dose-response curves with XLfit©.

### Analysis of effect of E3 ligase inhibitors on *P. falciparum* development (stage specific imaging analysis: antimalarial assay)

The stage-specific activity of the lead compounds was tested against *Pf* D6. The parasite culture was grown in RPMI-1640 medium supplemented with amikacin (60 μg/mL), NaHCO_3_ (27 mM) and 10% heat-inactivated normal human type A+ serum. The culture was synchronized by 5% sorbitol treatment as previously described [16]. The synchronized culture of *Pf*D6, with ~6% parasitemia and >90% of the parasite in ring stage, was used for these assays. Stock solutions (10 mM) of the test inhibitors were prepared in DMSO. CQ and active antimalarial inhibitors namely, Nutlin 3, JNJ 26854165 and HLI 737 were tested at 50 nM, 35 μM, 5 μM and 5 μM concentration respectively for the stage-specific antimalarial assay. These concentrations were almost equivalent to the IC_90_ of the inhibitors against *PfD6* at 48 h. The effect of the compounds was monitored after every 8 h (0, 8, 16, 24, 32, 40 and 48 h) by preparing thin smears of the infected erythrocytes. The slides were stained with Giemsa, and digital images of the parasite were captured with Nikon Eclipse microscope under bright field using NIS-element imaging software. The images were analyzed for the parasite stages and morphological changes (if any) in the parasite. The levels of parasitemia and differential parasitemia (relative percentage of different parasite stages) counts were also measured. At least one thousand erythrocytes (total- infected & uninfected) were counted from each slide for determination of the the level of parasitemia and differential parasite stage counts.

### Statistical analysis

Two-way ANOVA followed by Tukey and Sidak multiple comparison test with GraphPad Prism 6 was used to compare IC_50_ values of the inhibitors, the parasitemia and also percentage of individual parasite stages.

## Results

### In vitro antiplasmodial activity of standard E3 ubiquitin ligase inhibitors

**Fig. 1:**
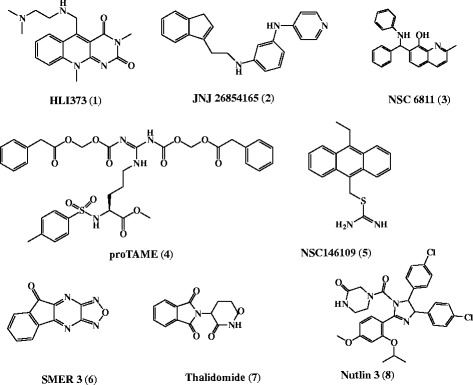
Chemical structures of the E3 ubiquitin ligase inhibitors. Eight E3 ligase inhibitors tested in vitro for antimalarial activity against CQ sensitive *P. falciparum* D6 strain (*Pf*D6) and CQ resistant *P. falciparum* W2 strain (*Pf*W2)

The antimalarial activity of a set of E3 ubiquitin ligase inhibitors was evaluated in vitro against *Pf*D6 and *Pf*W2 at eight different concentrations of each compound and at four different time points (48, 72, 96 and 120 h) (Table 1). Among the eight E3 ligase inhibitors tested (Fig. 1), thalidomide and proTAME did not show noticeable antimalarial activity against *Pf*D6 and *Pf*W2 up to 50 μM concentration and 120 h of exposure. E3 ligase inhibitors, namely NSC 66811, SMER 3 and Nutlin 3 showed moderate antimalarial activity against both *Pf*D6 & *Pf*W2. Antimalarial activity of above three inhibitors against *Pf*D6 was not significantly different from the activity against *Pf*W2 except SMER 3. SMER 3 showed significantly better antimalarial activity at 72 h (*p* < 0.05) against *Pf*W2 as compared to *Pf*D6. NSC 146109, JNJ 26854165 and HLI 373 showed lower IC_50_ values (below 6 μM) against both *Pf*D6 and *Pf*W2 and exhibited early growth inhibition. The IC_50_ values of HLI 373 against *Pf*D6 were significantly different and were almost 2 fold higher against *Pf*W2 as compared to *Pf*D6 at 48 (*p* < 0.05), 72 (*p* < 0.01), 96 (*p* < 0.01) and 120 h (*p* < 0.01). The antimalarial activity of JNJ 26854165 against *Pf*D6 was significantly higher (lower IC_50_ values) in comparison to *Pf*W2 at 72 h (*p* < 0.05). Similarly, NSC 146109 showed significantly better antimalarial activity against *Pf*W2 as compared to *Pf*D6 at 48 (*p* < 0.0001) and 72 h (*p* < 0.001).

**Table 1:** In vitro antimalarial activity of E3 ubiquitin ligase inhibitors

Compound Name	*P. falciparum* D6 (CQ-sensitive strain) IC_50_ (μM)				*P. falciparum* W2 (CQ-resistant strain) IC50 (μM)				Vero Cell Cytotoxicity ^a^	THP1 Cell Cytotoxicity ^d^
	48 h	72 h	96 h	120 h	48 h	72 h	96 h	120 h	48 h	48 h
Chloroquine	0.03 ±0.001	0.02 ±0.004	0.02 ±0.001	0.02 ±0.001	0.19 ±0.004^b^ ****	0.19 ±0.007 ^b^ ***	0.20 ±0.001 ^b^ ****	0.19 ±0.002 ^b^ ***	NC	NC
Thalidomide	NA	NA	NA	NA	NA	NA	NA	NA	NC	NC
proTAME	NA	NA	NA	NA	NA	NA	NA	NA	NC	NC
NSC 66811	32.18 ±4.57	37.02 ±1.80	NA	NA	28.74 ±4.01	38.95 ±2.71	NA	NA	NC	37.8 ±4.1
Nutlin 3	12.76 ±1.34	19.43 ±0.34	15.93 ±1.24	20.02 ±4.52	18.56 ±1.6	15.72 ±0.04	15.44 ±0.54	15.59 ±2.17	NC	35.2 ±2.76^e^ ^f##^
HLI 373	2.36 ±0.05	3.07 ±0.04	2.98 ±0.01	2.43 ±0.25	3.47 ±0.02 ^b^ *	4.81 ±0.41 ^b^ **	4.74 ±0.09 ^b^ **	4.04 ±0.04 ^b^ **	NC	23.2 ±0.07 ^e^ ^f ###^
JNJ 26854165	2.17 ± 0.004	2.93 ± 0.03	1.41 ±0.03	1.46 ±0.12	1.86 ±0.03	1.74 ±0.27 ^b^ *	2.28 ±0.03	2.02 ±0.36	26.9 8 ± 1.96^c^	22.4 ±0.56 ^e^ ^f ###^
SMER 3	12.06 ± 0.68	49.04 ±0.94	NA	NA	20.58 ±5.19	23.69 ±3.53 ^b^ *	45.80 ±0.09	48.58 ±0.49	16.0 ±0.55	5.8 ±0.28 ^f #^
NSC 146109	5.24 ± 0.20	4.80 ±0.05	3.09 ±0.08	2.52 ±0.39	2.07 ±0.22 ^b^ ****	2.63 ± 0.05 ^b^ ***	2.91 ±0.03	3.23 ±0.03	5.27 ±0.05	1.2 ±0.28

Antimalarial activity (IC50 values in μM) of E3 ubiquitin ligase inhibitors and CQ against asexual blood stages of the *Pf*D6 and *Pf*W2 was determined from the dose-response inhibition with XLFit after 48, 72, 96 and 120 h of incubation

^a^Cytotoxicity (IC50 values in μM) of E3 ubiquitin ligase inhibitors and CQ against Vero cells after 48 h

^d^Cytotoxicity (IC50 values in μM) of E3 ubiquitin ligase inhibitors and CQ against THP1 cells after 48 h. The highest concentration used for E3 ligase inhibitors and CQ was 50 μM and 500 nM respectively. Values are mean ± SEM of duplicate observations. NA, not active at the highest concentration tested. NC, not cytotoxic at the highest concentration tested

^b^Statistically significant difference compared to corresponding activity against *Pf*D6 (* *p*-value <0.05; ** *p*-value <0.01; ***p-value <0.001; **** *p*-value <0.0001)

^c^Statistically significant difference (*p*-value <0.001) compared to corresponding activity against *Pf*D6 and *Pf*W2

^e^Statistically significant difference (*p*-value <0.001) compared to corresponding activity against *Pf*D6

^f^Statistically significant difference (^#^ *p*-value <0.05; ^##^ *p*-value <0.01; ^###^ *p*-value <0.001; ^####^ *p*-value <0.0001) compared to corresponding activity against *Pf*W2

### In vitro cytotoxicity of standard E3 ligase inhibitors against Vero and THP1 cells-

The E3 ligase inhibitors tested for in vitro antimalarial activity were also tested against Vero cells (African green monkey kidney epithelial cells) and non-proliferating THP1 (transformed human monocytic) cells for mammalian cell cytotoxicity (Table 1). CQ, thalidomide, proTAME, NSC 66811, Nutlin 3 and HLI 373 were not toxic at the highest concentration tested. SMER 3 and NSC 146109 were toxic against Vero cells, and their IC_50_ values were not considerably different from corresponding IC_50_ values against *Pf*D6 and *Pf*W2. However, JNJ-26854165 was significantly less toxic to mammalian cells as compared to *Pf*D6 (*p*-value <0.001) and *Pf*W2 (p-value <0.001). Therefore Nutlin 3, HLI 373 and JNJ-26854165 were identified as selective antimalarial E3 ligase inhibitors. CQ, thalidomide, and proTAME were not toxic against THP1 cells at the highest concentration tested. NSC 146109 and SMER 3 were toxic against THP1 cells, and IC50 values of NSC 146109 was not considerably different from corresponding IC50 values against *PfD6* and *PfW2*. The IC50 value of SMER 3 was not considerably different from corresponding IC50 values against *PfD6*, although significantly different than corresponding IC50 values against *PfW2* (*p*-value <0.05). NSC 66811 was not toxic against THP1 cells. Nutlin 3, HLI 373 and JNJ-26854165 were considerably less toxic against THP1 cells as compared to *PfD6* (*p*-value <0.001, <0.001 and <0.001 respectively) and *PfW2* (*p*-value <0.01, <0.001 and <0.001 respectively). Thus Nutlin 3, HLI 373 and JNJ-26854165 were identified as selective antimalarial E3 ligase inhibitors.

### Evaluation of antimalarial ubiquitin ligase inhibitors on growth and development of *P. falciparum*

**Fig. 2:**
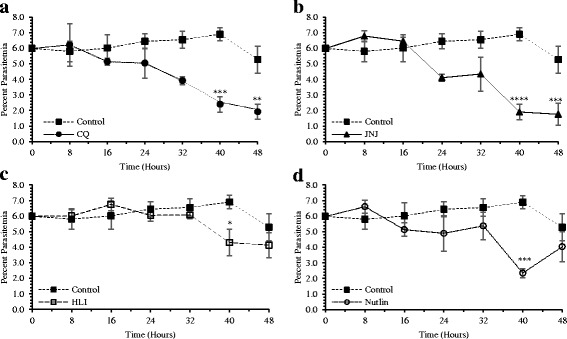
Effect of selected E3 ligase inhibitors on growth of *Plasmodium falciparum* in vitro*.* Each point represents the mean ± SEM of three values. Minimum one thousand erythrocytes were counted for each value. **a** Chloroquine (CQ) was tested as standard antimalarial drug for positive control and DMSO was used as vehicle control. The ring stage synchronized CQ sensitive *P. falciparum* D6 strain (*Pf* D6) culture with high parasitemia (~6%) was treated with the antimalarial E3 ligases inhibitors namely, **b** JNJ 26854165 (JNJ), **c** HLI 373 (HLI) and (D) Nutlin-3 (Nutlin). Parasitemia was monitored at 0, 8, 16, 24, 32, 40 and 48 h post treatment as indicated. Statistically significant difference compared to corresponding hours against vehicle control (* *p*-value <0.05; ** *p*-value <0.01; ***p-value <0.001; **** *p*-value <0.0001)

**Fig. 3:**
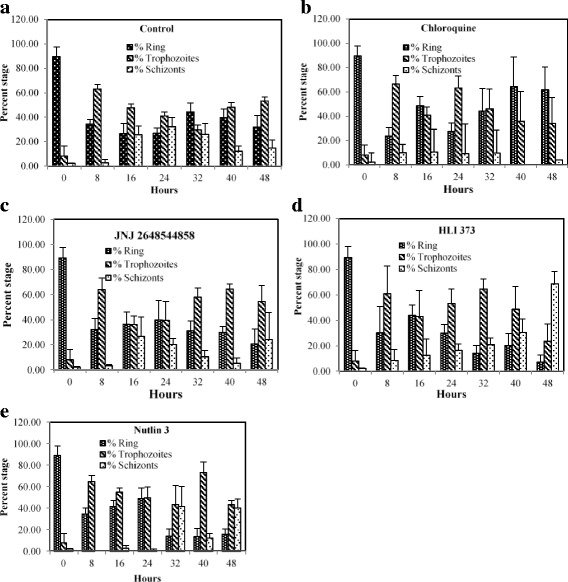
Effect of selected E3 ligase inhibitors on cellular development of *Plasmodium falciparum* in vitro. **a** Control [DMSO]; **b** Chloroquine; **c** JNJ 26854165; **d** HLI 373; **e** Nutlin-3 at 0, 8, 16, 24, 32, 40 and 48 h post-treatment. Each bar represents the mean ± SEM of three observations. Minimum one thousand erythrocytes were counted for each observation. Chloroquine (CQ) and DMSO were tested as positive, and vehicle control respectively. The ring stage synchronized CQ-sensitive *P. falciparum* D6 strain (*Pf* D6) culture with high parasitemia (~6%) was treated with the lead E3 ligase inhibitors and differential parasite stages were monitored at 0, 8, 16, 24, 32, 40 and 48 h as indicated. The results on differential parasite stage counts in control, CQ and inhibitors treated *P. falciparum* cultures were analyzed for statistical comparisons. The change in differential parasite counts in inhibitor treated cells at 48 hors post-treatment were statistically not significant (*P* values >0.5) compared to controls and CQ treated *Pf*D6 cultures due to large individual variations

**Fig. 4:**
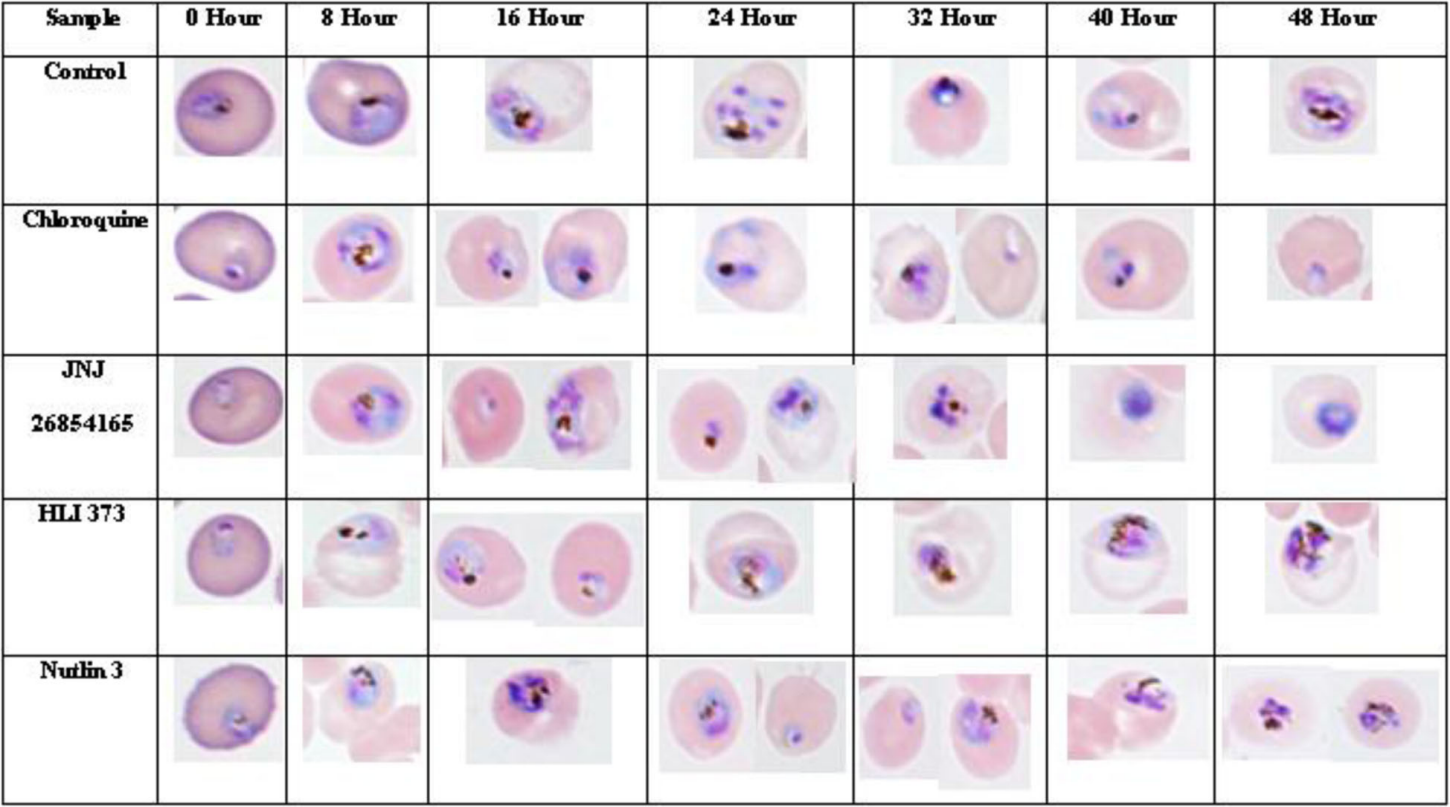
Progression of cellular development of asexual *Plasmodium falciparum* during treatment with antimalarial E3 ligase inhibitors. *P. falciparum* D6 strain (*Pf* D6) cultures were treated with vehicle control (DMSO), chloroquine, JNJ 26854165, HLI 373 and Nutlin-3 and digitally monitored at 0, 8, 16, 24, 32, 40 and 48 h’ post-treatment for parasite stages. Chloroquine (CQ) was used as positive control. Only selective digital images from each set of observations are represented here

The effects of lead antimalarial ubiquitin ligase inhibitors namely, JNJ 26854165, HLI 373 and Nutlin 3 were further tested against the asexual intra-erythrocytic development of *P. falciparum*. These inhibitors showed early effects on the parasite growth (Table 1) and therefore were tested up to 48-h incubation period. The results show the effects of inhibitors on overall levels of the parasitemia (Fig. 2), and relative percentages of different parasite stages namely rings, trophozoites and schizonts (Fig. 3). Any morphological and cellular changes in parasite development were determined by analysis of digital images of the parasite exposed to these inhibitors. The representative digital images of the parasite stages and modifications in parasite morphology are presented in Fig. 4. CQ was tested as a positive control, and DMSO was tested as a vehicle control in this assay. At 0 h the *Pf*D6 cultures had ~ 6% overall parasitemia and > 90% of the parasites were in ring stage. In *Pf*D6 culture treated with vehicle control majority of the parasites were transformed into trophozoites at 8 to 48 h, The ratio of schizonts increased from 0 to 24 h and declined at after 32 h (Fig. 3a). The overall level of parasitemia did not change considerably in vehicle treated *Pf*D6 culture up to 48 h, as compared to 0 h (5.28% vs. 5.98%) (Fig. 2). CQ treatment significantly decreased the overall parasitemia at 40 (*p* < 0.001) and 48 (*p* < 0.01) hours, as compared to the vehicle control (Fig. 2a). In CQ treated *PfD6* cultures, around 50% of the parasites were arrested at ring stage at 16 h post-treatment. Likewise, more than 50% of the parasites were arrested at ring stage at 40 and 48 h. Only a few parasites matured into schizonts, which further declined after 32 h (Fig. 3b). The change in differential parasite counts in CQ-treated cells, at 48 h post-treatment, were statistically not significant compared to control vehicle treated *Pf*D6 culture, due to large individual variations. However, the rings detected in CQ-treated *Pf*D6 cultures were apparently healthy, but the trophozoites were shrunk (Fig. 4). These results indicate that CQ kills the malarial cells by blocking the growth of parasite at ring/schizont stage. This reduction in parasitemia by JNJ 26854165 was comparable to that of CQ at 40 (1.90% vs 2.40%) and 48 h (1.76% vs 1.92%), and was markedly reduced as compare to control at 40 (1.9 vs 6.90; *p* < 0.0001) and 48 (1.76% vs 5.28%; *p* < 0.01) hours of treatment (Fig. 2a and b). The parasites treated with JNJ 2648544858 apparently showed a different life-cycle stage development pattern as compared to control. The majority (~90% at 0 h) of the rings stage parasite matured to trophozoites at 8 h. At 16 and 24 h the majority of *P. falciparum* parasites were in ring and trophozoite stages, and again trophozoites became dominant at 40 and 48 h (Fig. 3c). The change in differential parasite counts in JNJ2648544858 treated cells at 48 h post-treatment were statistically not significant compared to controls and CQ-treated *Pf*D6 cultures due to large individual variations (Fig. 3). However, distorted rings, trophozoites, and schizonts were seen in *Pf*D6 cultures treatment with JNJ 2648544858 at 40 and 48 h post-treatment (Fig. 4). HLI 373 showed a significant reduction in parasitemia at 40 h (*p* < 0.05) as compared to control (Fig. 2c). The *P. falciparum* culture treated with HLI 373 showed ring stages in the majority (~90%) at 0 h, while at the remaining time points after the treatment (8, 16, 24, 32 and 40) majority of the parasite cells were at trophozoite stage. Most of the parasite cells were at schizont stage at 48 h after the treatment with HLI 373 (Fig. 3d). However, the change in differential parasite counts in HLI 373 treated cells at 48 h post-treatment were statistically not significant compared to controls and CQ-treated *Pf*D6 cultures due to large individual variations (Fig. 3). The parasitemia was significantly reduced in Nutlin 3 treated *P. falciparum* culture as compared to control at 40 h (*p* < 0.001) (Fig. 2d). In *P. falciparum* cultures treated with Nutlin 3 trophozoites were in the majority at 8, 16 and 40 h post-treatment, whereas trophozoites and schizonts were the dominating stages at 32 and 48 h post-treatment (Fig. 3e). The change in differential parasite counts in Nutlin 3 treated cells at 48 h post-treatment were statistically not significant compared to controls, and CQ-treated *Pf*D6 cultures due to large individual variations (Fig. 3). However, A large number of distorted trophozoites and schizonts were observed in HLI 373, and Nutlin 3 treated *P. falciparum* culture at 40 and 48 h post-treatment (Fig. 4).

## Discussion

The ubiquitin system of *P. falciparum* is involved in numerous cellular functions and host-pathogen interaction [3]. The functions of ubiquitin system are essential for progression of the malaria parasites’ life cycle [3]. The *P. falciparum* genome contains more than 100 genes, which putatively encode for the component associated with the ubiquitin system. The *P. falciparum* ubiquitin system contains four ubiquitins (PFL0585w, PF14_0027, PF13_0346, and PF08_0067), eight ubiquitin-activating enzymes (specific for ubiquitin and ubiquitin-like moieties), 14 ubiquitin-conjugating enzymes and more than fifty E3 ubiquitin-protein ligases. The proteomic analysis of *P. falciparum* ubiquitome has shown that the level of ubiquitin was more in rings and trophozoites than in schizonts [4], which suggested that ubiquitylation of proteins occurred more prominently in schizonts’ and might be necessary for schizonts’ maturation and their further differentiation into invasive merozoites [4].

Among the components of ubiquitin pathway, E3 ubiquitin-protein ligase is the most variable and divergent within eukaryotes and highly specific to the *P. falciparum* parasite [4]. Thus, targeting essential parasite-specific E3 ligases would be a promising approach for molecular target-based new antimalarial drug discovery [5]. E3 ubiquitin-protein ligase catalyzes the transfer of activated ubiquitin to the specific target protein. E3 ubiquitin-protein ligases are divided into three main classes on the basis presence of specific domains namely, Homologous to E6-associated protein C-terminus (HECT), Really Interesting New Gene (RING) and U-box [3]. The standard E3 ligase inhibitors tested in this study showed an early antimalarial effect after the treatment, which was consistent up to 120 h. This suggests that these inhibitors kill the parasites in the first asexual cycle. However, some classes of drugs – specifically, antibiotics like chloramphenicol, clindamycin, rifampin, tetracycline, telithromycin and quinupristin-dalfopristin show delayed killing of the malaria parasite in the second cycle of parasite’s asexual reproduction [17]. These antibiotics inhibit the protein synthesis in general, and the primary target of antibiotic antimalarials in *Plasmodium sp.* seems to be in the apicoplast. By inhibiting protein synthesis and the apicoplast, these antibiotics cause a delayed death phenomenon [17–20].

The E3 ligase inhibitors namely, JNJ 26854165, HLI 373 and Nutlin 3 were found to be active against both CQ-sensitive *Pf*D6 and CQ-resistant *Pf*W2 and were less cytotoxic against Vero and transformed THP1 cells. The two E3 ligase inhibitors namely, JNJ 26854165 and HLI 373 are HDM2 antagonists. HDM2 is a RING finger E3-ubiquitin ligase that acts as the predominant negative regulator of p53 [10, 21–23]. JNJ 26854165, also known as Serdemetan, is a tryptamine derivative and interrupts the HDM2-p53 binding and is in phase 1 clinical trial for the treatment of multiple myelomas [21, 22, 24]. Nutlin 3 is an inhibitor of MDM2 (Murine Double Minute 2), an oncogene and a negative regulator of the tumor suppressor protein p53. Nutlin 3 blocks MDM2-p53 interaction with potency in sub-nanomolar range with Ki value 36 nM [10, 25]. Like Nutlin 3, NSC 66811 also is an MDM 2 inhibitor with Ki value 120 nM but showed less prominent activity against *Pf*D6 and *Pf*W2 as compare to Nutlin 3 [11]. ProTAME is a cell-permeable prodrug, which is converted to its active parent molecule TAME. The parent molecule inhibits the ubiquitin ligase activity of the APC/C by preventing its activation by Cdc20 and Cdh1 [8, 9].

The antimalarial active E3 ligase inhibitors arrested the parasite growth at trophozoites stages resulting in accumulation of distorted trophozoites and immature schizonts. These observations are in agreement with previous reports [5], which showed more predominant expression of E3 ligase in trophozoites and early schizonts. The ubiquitin/proteasome function may be essential for maturation of *P. falciparum* trophozoites.

## Conclusions

E3 ligase inhibitors namely, JNJ 26854165, HLI 373 and Nutlin 3 showed prominent antimalarial activity against both CQ- sensitive *Pf*D6 and -resistant *Pf*W2 strains. JNJ 26854165 showed considerably different activity against *Pf*D6 as compared to *Pf*W2 at 72-h post treatment. Similarly, the antimalarial activity of HLI 373 was considerably different against *Pf*D6 as compared to *Pf*W2 at 48, 72, 96 and 120 h. However, Nutlin 3 did not show considerably different activity against *Pf*D6 as compared to *Pf*W2 at all four-time points. The inhibitors namely, JNJ 26854165, HLI 373 and Nutlin 3 were selectively active against *P. falciparum*, and did not show cytotoxicity against Vero and transformed THP1 cells. JNJ 26854165, HLI 373 and Nutlin 3 apparently arrest the development of *P. falciparum* growth at the trophozoites and schizonts stage and cause accumulation of distorted trophozoites and immature schizonts.

These results suggest that the function of ubiquitin/proteasome may be crucial for schizont maturation in *P. falciparum* parasite. Antimalarial E3 ligase inhibitors apparently block trophozoites and schizonts maturation and inhibit the intraerythrocytic development of malaria parasite. Further investigations on the lead antimalarial E3 ligase inhibitors would promise new understanding and importance of E3 ligase function in the malaria parasite. It may also provide a viable antimalarial-target and new classes of antimalarial drug leads.

## Abbreviations

APC/C: Anaphase-promoting complex/cyclosome
CQ: Chloroquine
CRBN: Cereblon
Cul4A: Cullin-4A
DDB1: Damaged DNA binding protein 1
DMSO: Dimethyl sulfoxide
FBS: fetal bovine serum
HDM2: Human double minute 2
HECT: Homologous to E6-associated protein C-terminus
MDM2: Murine double minute 2
*Pf* D6: Chloroquine-sensitive *Plasmodium falciparum* D6 strain
*Pf* W2: Chloroquine -resistant *Plasmodium falciparum* W2 strain
RING: Really interesting new gene
TAME: Tosyl-L-arginine methyl ester
